# Splenic rupture after video-assisted thoracoscopic lobectomy: a case report

**DOI:** 10.1093/jscr/rjaf240

**Published:** 2025-07-28

**Authors:** Xibei Hu, Huixiang Xu, Yanhu Xie

**Affiliations:** Department of Anesthesiology, Division of Life Science and Medicine, The First Affiliated Hospital of USTC (Anhui Provincial Hospital), University of Science and Technology of China, Hefei, Anhui 230036, China; Department of Anesthesiology, Division of Life Science and Medicine, The First Affiliated Hospital of USTC (Anhui Provincial Hospital), University of Science and Technology of China, Hefei, Anhui 230036, China; Department of Anesthesiology, Anhui Chest Hospital, Hefei, Anhui 230001, China; Department of Anesthesiology, Division of Life Science and Medicine, The First Affiliated Hospital of USTC (Anhui Provincial Hospital), University of Science and Technology of China, Hefei, Anhui 230036, China

**Keywords:** video-assisted thoracoscopic surgery (VATS), splenic rupture, E-FAST ultrasound, case report

## Abstract

This report details a rare splenic rupture following left video-assisted thoracoscopic lobectomy in a 50-year-old male. Intraoperative tachycardia (78 → 110 bpm) and postoperative refractory hypotension (56/33 mmHg) progressed to hemorrhagic shock (Hb 57 g/L) at 90 min post-op. E-FAST confirmed splenic rupture with hemoperitoneum, leading to emergent splenectomy for a 3-cm diaphragmatic surface laceration (3000 mL blood loss). Unique features include: (i) Traction-mediated injury despite intact diaphragm; (ii) Diagnostic delay from nonspecific early signs. This case underscores the need for vigilance regarding remote organ injury in minimally invasive thoracic surgery, advocating systematic protocols to manage this lethal complication.

## Introduction

Video-assisted thoracoscopic surgery (VATS) has been widely used for the diagnosis and treatment of thoracic diseases since the 1990s. The complication rates of VATS are generally very low, with common issues including prolonged air leak, intrathoracic hemorrhage, infection, and postoperative pain [1]. In this paper, we report a case of splenic rupture secondary to VATS, which is a very rare but serious and potentially catastrophic event. This report adheres to the Surgical CAse REport (SCARE) criteria [2].

## Case report

A 50-year-old male (body mass index, 23.5 kg/m^2^) was admitted for evaluation of a left lower pulmonary nodule detected during routine examination, ultimately undergoing thoracoscopic wedge resection. His medical history included well-controlled type 2 diabetes managed with metformin (850 mg twice daily), acarbose (50 mg three times daily), and dapagliflozin (10 mg daily). Preoperative evaluation revealed hemoglobin (Hb) 126 g/L, hematocrit (Hct) 39%, mild mitral regurgitation on echocardiography, and unremarkable abdominal ultrasonography findings.

Pre-induction non-invasive blood pressure was 129/ 80 mmHg, heart rate (HR) was 80 bpm, respiratory rate was 18 breaths per minute, peripheral oxygen saturation was 99%, and the bispectral index valued 100. Anesthesia was induced with glycopyrrolate 0.5 mg, dexamethasone 5 mg, etomidate 14 mg, remimazolam 10 mg, sufentanil 30 μg, and rocuronium 50 mg. Following successful placement of a right-sided double-lumen endotracheal tube (37F, 29 cm depth) under bronchoscopic guidance, anesthesia maintenance involved continuous infusion of ciprofol (0.4–0.8 mg/kg/h) and remifentanil (0.05–0.15 μg/kg/min) with 1% sevoflurane, supplemented by cisatracurium (0.05 mg/kg/h).

Upon establishing right lateral decubitus positioning for one-lung ventilation, progressive tachycardia developed (HR 78 → 110 bpm) alongside blood pressure fluctuations (115 → 100/70 → 55 mmHg). Despite implementing analgesia optimization, fluid resuscitation (800 mL balanced crystalloid), ventilation parameter adjustments, dexmedetomidine infusion, and intermittent esmolol/phenylephrine administration, hemodynamic stabilization remained suboptimal. The wedge resection was completed within 20 min, after which intraoperative frozen analysis necessitated conversion to left lower lobectomy. Total procedural duration was 40 min with minimal blood loss (10 mL).

**Figure 1:**
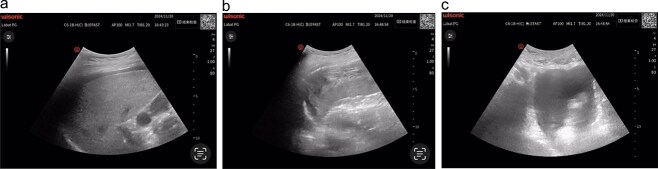
(a) Hepatic ultrasonography demonstrates an intact hepatic capsule with adjacent anechoic fluid collections; (b) splenic imaging reveals discontinuous capsular contour at the diaphragmatic surface with heterogeneous parenchymal echotexture; (c) pelvic cavity shows significant anechoic fluid accumulation.

Following supine repositioning, the patient developed hypotension (74/38 mmHg) that transiently responded to phenylephrine bolus and 500 mL colloid infusion, with blood pressure stabilizing at 109/69 mmHg prior to transfer to the post-anesthesia care unit (PACU). However, within 5 min of PACU arrival, profound hemodynamic collapse occurred (56/33 mmHg, HR 94 bpm) refractory to repeated norepinephrine administration. Immediate arterial blood gas analysis revealed metabolic acidosis (pH 7.299, base excess −6.4 mmol/L) concurrent with acute anemia (Hb 77 g/L). Emergency E-FAST subsequently identified splenic capsular discontinuity and significant hemoperitoneum (Fig. 1), confirmed by ultrasound-guided aspiration of non-clotting blood. Hemoglobin levels progressively declined to a nadir of 57 g/L during this critical phase.

After confirmation of intra-abdominal hemorrhage (suspected splenic rupture), emergent exploratory laparotomy was performed, revealing a 3-cm actively bleeding laceration along the splenophrenic ligament's adhesion band on the diaphragmatic surface. Splenectomy was subsequently conducted with 3000 mL intraoperative blood loss and 500 mL urine output. Fluid resuscitation included 2000 mL balanced crystalloid, 500 mL hydroxyethyl starch, 3 units leukocyte-reduced packed RBCs, 375 mL fresh frozen plasma, and 500 mL cell-salvaged autologous blood. Postoperative blood gas analysis prior to Intensive Care Unit transfer showed Hb 70 g/L and Hct 21.3%. Following 48-h intensive monitoring, the patient transitioned to ward care and was discharged on postoperative day 15.

## Discussion

Splenic rupture represents an exceptionally rare yet critical perioperative complication in thoracoscopic procedures. Previous case reports [3–5] describe similar clinical scenarios where patients developed abdominal pain and/or hemodynamic instability during post-anesthesia recovery, subsequently confirmed by CT or ultrasonography. Notably, only one documented case achieved intraoperative diagnosis through point-of-care E-FAST ultrasound when acute hemodynamic instability, abdominal distension, and precipitous hemoglobin decline occurred during surgery, highlighting the diagnostic superiority of bedside ultrasonography in acute thoracoabdominal trauma. In the present case, intraoperative diagnosis was complicated by the absence of blood gas analysis and the nonspecific early presentation of tachycardia, while blood pressure maintenance through fluid resuscitation and intermittent vasopressors could be attributed to multiple etiologies. Moreover, the predominant association of hemorrhagic shock with intrathoracic origins in such procedures further delayed recognition. The diagnostic breakthrough emerged postoperatively when severe hypotensive shock prompted urgent blood gas analysis revealing critical anemia (Hb 57 g/L), followed by bedside radiography and FAST ultrasound demonstrating splenic capsular disruption with massive hemoperitoneum, ultimately confirmed by exploratory laparotomy.

A systematic review of thoracoscopy-associated splenic injuries reveals three consistent features: (i) Exclusive occurrence in left thoracic procedures; (ii) Predilection for upper pole involvement with temporal presentation spanning intraoperative to 72 h postoperatively, ranging from subcapsular hematomas managed conservatively to frank rupture requiring surgery; (iii) Intact diaphragmatic integrity despite potential intraoperative blunt traction forces. Our case aligns with these characteristics but demonstrates unique biphasic progression: initial subcapsular hematoma formation during surgery evolving into complete capsular rupture following positional changes. Anatomically, the spleen’s vulnerable position—nestled in the left upper quadrant beneath the diaphragm, separated from the left thoracic cavity only by the diaphragmatic muscle, and anchored by four ligaments (gastrosplenic, splenorenal, splenocolic, and splenophrenic)—predisposes it to iatrogenic injury through direct/indirect ligament traction. While such injuries more commonly occur in abdominal surgeries (e.g. gastrectomy, colonoscopy) [6, 7], our operative findings of a 3-cm laceration at the splenophrenic ligament adhesion band, without diaphragmatic perforation, strongly support the mechanism of diaphragmatic traction-induced capsular tear. Beyond this primary mechanism, non-traumatic rupture predisposing factors including sudden increases in intra-abdominal pressure (e.g. sneezing, coughing, defecation) [8] warrant consideration in differential diagnosis.

This case underscores three critical imperatives for anesthesiologists: (i) Protocol-driven monitoring: Implementation of guideline-recommended invasive hemodynamic monitoring for high-risk thoracic procedures; (ii) Diagnostic agility: Maintenance of diagnostic vigilance when conventional interventions prove ineffective, necessitating immediate multidisciplinary consultation and utilization of rapid-assessment ultrasound techniques (e.g. E-FAST, TTE); (iii) Systems preparedness: Establishment of institutional protocols integrating anesthesia-surgery-radiology collaboration for time-sensitive decision-making. Through these measures, clinicians can enhance early detection of occult injuries and optimize management of such life-threatening complications.
